# Prioritization in medical school simulation curriculum development using survey tools and desirability function: a pilot experiment

**DOI:** 10.1186/s41077-018-0061-x

**Published:** 2018-02-26

**Authors:** Pier Luigi Ingrassia, Ludovico Giovanni Barozza, Jeffrey Michael Franc

**Affiliations:** 1 Centro Interdipartimentale di Didattica Innovativa e di Simulazione in Medicina e Professioni Sanitarie”–SIMNOVA, Università del Piemonte Orientale, via Lanino 1, 28100 Novara, Italy; 2 Department of Emergency Medicine, 790 University Terrace Building, 8303-112 Street, Edmonton, AB T6G 2T4 Canada

**Keywords:** Education, Curriculum design, Technical skill, Simulation, Medical students

## Abstract

**Background:**

In Italy, there is no framework of procedural skills that all medical students should be able to perform autonomously at graduation. The study aims at identifying (1) a set of essential procedural skills and (2) which abilities could be potentially taught with simulation. Desirability score was calculated for each procedure to determine the most effective manner to proceed with simulation curriculum development.

**Methods:**

A web poll was conducted at the School of Medicine in Novara, looking at the level of expected and self-perceived competency for common medical procedures. Three groups were enrolled: (1) faculty, (2) junior doctors in their first years of practice, and (3) recently graduated medical students. Level of importance of procedural skills for independent practice expressed by teachers, level of mastery self-perceived by learners (students and junior doctors) and suitability of simulation training for the given technical skills were measured. Desirability function was used to set priorities for future learning.

**Results:**

The overall mean expected level of competency for the procedural skills was 7.9/9. Mean level of self reported competency was 4.7/9 for junior doctors and 4.4/9 for recently graduated students. The highest priority skills according to the desirability function were urinary catheter placement, nasogastric tube insertion, and incision and drainage of superficial abscesses.

**Conclusions:**

This study identifies those technical competencies thought by faculty to be important and assessed the junior doctors and recent graduates level of self-perceived confidence in performing these skills. The study also identifies the perceived utility of teaching these skills by simulation. The study prioritizes those skills that have a gap between expected and observed competency and are also thought to be amenable to teaching by simulation. This allows immediate priorities for simulation curriculum development in the most effective manner. This methodology may be useful to researchers in other centers to prioritize simulation training.

## Introduction

Theoretical knowledge and clinical skills are two equally important parts of medical education [1]. While many authorities agree on the importance of acquisition of technical skills, few guidelines detail the particular competencies medical students ought to acquire prior to graduation. Some studies suggest that the competencies among graduates may be highly variable, and that medical schools are not always effective in teaching important procedurals skills [2, 3]. In Italy, there is no published list of competencies for basic technical skills. In the 32 points of the “Qualifying Educational Goals” chart published by the *Ministry of Education, University and Research* (MIUR) in 2007, non-technical skills are widely discussed and recommended, but technical skills are only vaguely addressed [4]. To the authors’ knowledge, only Vettore et al. attempted to identify the technical skills which should be included in the medical school core curriculum [5].

The effectiveness and utility of simulation-based medical educations (SBME) has been well documented [2]. Simulation training offers opportunity for learners to practice both technical and non-technical skill in a low-risk setting. Learners can learn from both success and errors without the untoward patient risks [6–10]. As simulation technology and experience advance, the breadth of skills that can be simulated continues to widen. Simulation offers an opportunity previously unavailable for safe practice of psycho-motor and technical skills at any level of expertise.

Thus, although simulation may be a valuable tool for acquisition of technical skills, it may be difficult for medical schools to identify how to proceed in developing a simulation curriculum. Simulation can be costly in terms of staff, resources, simulation equipment, and giving learners time away from their clinical duties. It is unlikely that most medical schools will be able to immediately provide simulation training for all clinical skills. With no clear published guidelines on how to prioritize simulation training, it may be difficult to form an effective curriculum.

The objectives of this study were to (1) list technical skill competencies that medical students should possess prior to graduation, (2) determine the perceptions of faculty and learners about suitability of simulation training for technical skills, and (3) provide a prioritized list of these competencies for further curriculum development. It is the hope that curriculum designers will find precious tools they could benefit from.

## Methods

### Study design and population

The study is prospective observational study using a web-based survey tool. Three distinct groups participated in the study:

1. Faculty at the School of Medicine at the Università del Piemonte Orientale;
2. Junior doctors graduated in our medical school with 1–2 years clinical experience at the time of the study;
3. Medical students having successfully graduated but have not worked yet.

The study included all faculty members involved in teaching skills in the in the 2014–15 academic year, all medical students graduating in the same academic year, and all junior doctor with 1 or 2 years of clinical experience. An e-mail invitation including a brief presentation of the study, a consent form, and a link to the online questionnaire was sent to each participant. A maximum of three reminder e-mails were further forwarded to non-responders from September 2015 through January 2016. Each participant could answer the poll only once.

Participation was voluntary. Respondents were assured confidentiality of their responses and received no financial incentives. As the study results were presented in aggregate with no identifiers, the study was deemed exempt from formal institutional approval by the local ethics committee (Comitato Etico Interaziendale, Novara, Italy; study number Prot. 639/CE).

### Survey tool

The initial list of technical procedures was created by a brief review of current national and international literature. The list was intended to be comprehensive and inclusive. The survey was administered using SurveyMonkey (SurveyMonkey, Palo Alto, California, USA). The survey given to the faculty consisted of two main sections: the first rating their perception of the importance of each of the skills for independent practice, and the second rating their perception of the suitability of each skill for teaching by simulation. The survey for the junior doctors and students also consisted of two sections: the first rating their self-perceived mastery of the skill, and the second rating their perception of the suitability of the skill for teaching by simulation. In all cases, participants used a semantic differential scale for importance from 1 to 9 (1 being the least important and 9 being the most important). All participants were also given opportunity to add further skills that they felt should be included in the list. A detailed description of the survey instrument is provided in Table 1.

**Table 1:** Detailed description of the survey instrument

		Faculty	Graduating medical students	Junior doctors
Section 1	Question	How much essential is for student at the end of medical school to perform the following abilities in an autonomous and automatic manner?	How able you are in performing the following abilities in an autonomous and automatic manner?	After graduation, how much did you feel able to perform the following abilities in an autonomous and automatic manner without additional training?
	Linear numeric scale	From 1, absolutely not essential to 9, absolutely essential	From 1, absolutely not autonomous to 9, absolutely autonomous	From 1, absolutely not able to 9, absolutely able
	Aim	To estimate level of consensus about the importance over the selected procedural skills	To estimate the student actual level of mastery of the selected technical skills	To assess procedural skill needs in the clinical practice and limit bias of student sample selection
		(essentialness)	(autonomy)	(autonomy)
Section 2	Question	Would it be useful to teach the following abilities with the use of simulation before the clinical rotations?	Would it be useful to learn the following abilities with the use of simulation before the clinical rotations?	Would it be useful to teach the following abilities with the use of simulation?
	Linear numeric scale	From 1, absolutely useless to 9, absolutely useful	From 1, absolutely useless to 9, absolutely useful	From 1, absolutely useless to 9, absolutely useful
	Aim	To estimate the benefit of teaching those procedural skills before the clinical rotation, possibly by the use of simulation (utility)		
Section 3	Question	Would you like to list any additional procedures that you feel to be important but were omitted from the initial list?	Would you like to list any additional procedures that you feel to be important but were omitted from the initial list?	Would you like to list any additional procedures that you feel to be important but were omitted from the initial list?
	Aim	To include any additional procedures that were felt to be important but omitted from the initial list.		

### Definitions

The following terms are used and measured in the manuscript:

“essentialness” which estimates the level of faculty consensus about the importance that students at the end of medical school perform certain abilities in an autonomous and automatic manner;

“autonomy” which expresses the current level of mastery of these technical skills immediately after the graduation and after at least 1 year of experience in clinical setting for medical students and junior doctors respectively; and “utility” which refers to perceived suitability of simulation training for the given technical skills.

### Data analysis

Data from SurveyMonkey were transcribed to a spreadsheet using Microsoft Excel (Version 2003, Microsoft Corporation, Redmond, WA, USA). Data analysis was performed using *R: A Language and Environment for Statistical Computing* (R Foundation for Statistical Computing, Vienna, Austria). Data are presented as mean and interquartile range (IQR).

Desirability functions are well documented and frequently used in science and engineering when several responses must be optimized simultaneously [11]. These functions serve to transpose a multiple response problem into a single response problem [11]. In this study, the desirability function is written to deem to be of highest priority those abilities that (a) were thought to be very important to faculty (high score for autonomy), (b) were felt to be lacking in confidence for the recent graduates and junior doctors (low score for autonomy), and were believed teachable by simulation by all three groups (high score for utility). Desirability functions are usually developed by standardizing the value for each response over a scale of 0 to 1 and then taking the geometric mean of the response [11]. The final desirability function was the following:

For the faculty group:

$$ d=\sqrt{\left(\frac{\mathrm{utility}-1}{8}\right)\kern0.5em \left(\frac{\mathrm{autonomy}-1}{8}\right)} $$

For both the junior doctor group and the student group:

$$ d=\sqrt{\left(\frac{\mathrm{utility}-1}{8}\right)\kern0.5em \left(\frac{9-\mathrm{autonomy}}{8}\right)} $$

Finally, the overall desirability was calculated by combining the desirability scores from each of the three groups:

$$ {D}_{\mathrm{overall}}=\sqrt[3]{d_{\mathrm{teachers}}\kern0.5em \bullet \kern1em {d}_{\mathrm{junior}}\kern0.5em \bullet \kern1em {d}_{\mathrm{students}}} $$

This function gives results ranging from 0 to 1, where 0 is the lowest priority and 1 is the highest priority.

## Results

In total, 198 participants were invited to complete the survey: 43 faculty, 102 junior doctors, and 53 students. Overall, 132 agreed to participate (response rate 66.7%). The overall mean level of importance for each of the skills by group is presented in Table 2. Among the faculty, the skills rated most important (essentialness) were personal protective equipment (PPE) usage (9/9), venous puncture (8.9/9), intramuscular injection (9.9/9), and subcutaneous injection (8.9/9). Among junior doctors, ratings for self-perceived competency (autonomy) ranged from a high of 7.9/9 for personal protective equipment (PPE) usage to a low of 1.1/9 for cricothyrotomy. Recent graduates showed the same skills as highest for autonomy (7.6/9 for self-protection) and lowest for autonomy (1.3/9 for cricothyrotomy). Overall mean rating among all competencies for autonomy was 4.6 for junior doctors and 4.4 for recently graduated students.

**Table 2:** List of identified procedural skills and related results

			Section 1			Section 2		
			Essentialness	Autonomy		Utility		
n.	Procedural skills	Desirability score	Faculty	Students	Junior doctors	Teachers	Students	Junior doctors
#1	Urinary catheter placement and removal in males and females	0.77	7.6 (2.2)	2.2 (1.7)	2.5 (2.1)	7.8 (2.1)	7.9 (2.0)	7.8 (2.0)
#2	Nasogastric tube insertion and removal	0.75	8.1 (1.4)	2.3 (2.0)	2.7 (2.1)	7.6 (1.9)	7.4 (1.9)	7.1 (2.5)
#3	Superficial abscesses incision and drainage	0.70	7.1 (1.5)	2.3 (1.9)	2.9 (2.4)	7.8 (1.8)	7.4 (2.2)	7.0 (2.6)
#4	Pleural tap	0.69	6.1 (2.2)	1.6 (1.5)	1.9 (1.9)	7.0 (2.1)	7.1 (2.1)	6.5 (2.8)
#5	Lumbar puncture	0.69	5.6 (2.7)	1.3 (1.0)	1.3 (0.9)	7.0 (2.4)	6.9 (2.2)	6.2 (3.0)
#6	Cricothyrotomy	0.68	5.4 (2.6)	1.3 (0.9)	1.1 (0.5)	6.7 (2.8)	6.8 (2.4)	6.8 (2.8)
#7	Paracentesis	0.67	6.6 (2.5)	2.0 (1.9)	2.6 (2.3)	6.7 (2.6)	7.4 (2.0)	6.4 (2.7)
#8	Chest tube insertion and removal	0.65	5.3 (2.4)	1.7 (1.3)	1.8 (1.7)	6.6 (2.6)	7.3 (2.2)	6.9 (2.5)
#9	Nasal and oral intubation	0.65	6.0 (2.7)	2.9 (2.0)	2.4 (1.9)	7.3 (2.5)	7.4 (2.3)	7.2 (2.6)
#10	Continuous simple suture placement and removal	0.63	8.5 (1.0)	3.9 (2.7)	5.1 (3.1)	8.4 (1.5)	8.1 (2.0)	8.4 (1.4)
#11	First degree burn medication	0.63	8.3 (1.0)	2.9 (2.4)	4.8 (3.0)	7.4 (2.1)	7.4 (2.3)	7.0 (2.7)
#12	Ocular swab	0.62	6.7 (2.3)	2.6 (2.4)	3.1 (2.6)	6.5 (2.5)	6.9 (2.5)	5.9 (2.9)
#13	Polytraumatized patient immobilization for transport	0.61	8.6 (0.8)	5.1 (2.4)	4.2 (2.6)	8.5 (1.3)	7.9 (1.9)	7.7 (2.3)
#14	Venturi mask assembly and placement	0.61	7.8 (2.2)	4.2 (2.7)	3.9 (2.9)	7.3 (2.4)	7.4 (2.1)	7.2 (2.4)
#15	Superficial metal clips placement and removal	0.59	7.5 (1.8)	3.5 (2.6)	4.9 (2.9)	7.5 (2.6)	7.2 (2.4)	7.1 (2.4)
#16	Wound dressing placement	0.57	8.6 (0.8)	4.5 (2.4)	5.4 (2.8)	8.0 (1.9)	7.7 (2.3)	7.7 (2.3)
#17	Urethral swab	0.56	6.5 (2.3)	3.2 (2.6)	3.7 (2.9)	6.2 (2.5)	6.9 (2.4)	6.0 (2.8)
#18	Minor surgery surgical kit preparation	0.56	7.7 (1.3)	4.1 (2.4)	5.1 (2.5)	7.6 (2.0)	7.4 (2.2)	7.0 (2.3)
#19	Vaginal swab	0.55	6.7 (2.3)	3.6 (2.8)	4.0 (3.0)	6.6 (2.5)	6.9 (2.4)	6.0 (2.8)
#20	Spirometry	0.55	6.1 (2.1)	3.1 (2.4)	3.1 (2.5)	5.7 (2.6)	6.3 (2.6)	5.7 (2.9)
#21	Cervical collar placement	0.54	7.8 (2.0)	5.4 (2.5)	4.8 (2.7)	7.8 (2.3)	7.9 (1.8)	8.0 (1.8)
#22	Butterfly catheter placement and removal for venous puncture	0.54	8.9 (0.3)	5.3 (2.6)	5.4 (2.6)	8.1 (1.7)	7.7 (2.4)	7.8 (2.2)
#23	Radial artery puncture and blood sample	0.54	8.5 (0.8)	5.1 (2.9)	5.6 (2.5)	8.5 (1.1)	7.8 (2.0)	7.3 (2.3)
#24	Nasal swab	0.53	7.7 (1.6)	4.1 (2.9)	4.9 (3.0)	6.6 (2.4)	6.9 (2.4)	6.1 (2.8)
#25	Gynecologic exam	0.53	5.8 (3.0)	4.5 (2.5)	3.1 (2.7)	6.5 (2.8)	6.9 (2.5)	6.4 (2.6)
#26	Open wounds (cuts, sores, ulcers, and fistulas) cleansing and medication	0.53	8.7 (0.5)	4.7 (2.4)	6.0 (2.6)	7.6 (2.2)	7.7 (2.3)	7.4 (2.5)
#27	Bag valve mask (ambu bag) utilization	0.51	7.7 (2.1)	5.8 (2.7)	4.9 (3.2)	7.8 (2.3)	7.8 (2.1)	7.8 (2.0)
#28	Heimlich maneuver	0.50	8.6 (1.4)	6.0 (2.4)	5.9 (2.3)	8.6 (1.1)	8.3 (1.8)	8.4 (1.6)
#29	Pharingeal swab	0.49	7.6 (1.5)	5.0 (2.8)	5.0 (3.0)	6.8 (2.4)	7.0 (2.4)	6.0 (2.9)
#30	Inguinal canal examination	0.49	8.5 (1.0)	5.7 (2.5)	5.4 (2.9)	7.6 (1.9)	6.8 (2.7)	7.0 (2.6)
#31	Automated external defibrillator (AED) placement and utilization	0.48	8.7 (0.8)	6.1 (2.3)	6.3 (2.5)	8.6 (1.3)	8.4 (1.7)	8.6 (1.3)
#32	Cardiopulmonary resuscitation	0.43	8.7 (1.0)	6.6 (2.0)	6.6 (2.3)	8.7 (1.1)	8.5 (1.5)	8.7 (1.1)
#33	Glucometer utilization	0.42	8.7 (0.7)	5.9 (2.7)	6.6 (2.6)	7.4 (2.4)	6.9 (2.8)	6.9 (2.7)
#34	Intramuscular injection	0.40	8.9 (0.3)	6.5 (2.4)	6.8 (2.8)	7.9 (1.9)	7.4 (2.6)	7.4 (2.6)
#35	Subcutaneous injection	0.40	8.9 (0.3)	6.1 (2.5)	7.0 (2.6)	7.7 (2.0)	7.3 (2.7)	7.3 (2.7)
#36	Patient’s urine collection	0.37	8.4 (1.7)	6.5 (2.5)	6.7 (2.5)	6.8 (2.8)	7.0 (2.7)	6.7 (2.8)
#37	Peripheral blood smear	0.34	6.8 (2.5)	6.4 (2.3)	6.4 (2.4)	6.8 (2.6)	6.4 (2.7)	5.5 (2.8)
#38	Electrocardiograph placement and utilization	0.29	8.6 (1.4)	7.5 (1.9)	7.4 (2.2)	8.0 (1.8)	7.1 (2.6)	7.8 (2.3)
#39	Rectal exploration	0.29	8.5 (1.3)	7.4 (2.1)	7.5 (2.0)	7.9 (1.9)	6.9 (2.9)	7.2 (2.5)
#40	Personal protective equipment (PPE) usage	0.25	9.0 (0.2)	7.6 (2.0)	7.9 (1.9)	7.7 (2.4)	7.1 (2.9)	7.4 (2.7)
	Overall mean		7.64	4.42	4.67	7.44	7.34	7.08

NOTE: *Essentialness* refers to the level of importance of procedural skills which should be performed in an autonomous manner expressed by teachers, *Autonomy* refers to the level of mastery self-perceived by learners (students and junior doctors), *Utility* refers to the suitability of simulation training for the given technical skills before the clinical rotation

Desirability score reveals which abilities would be of highest priority to teach during simulation exercises (range from 0 to 1, where 0 is the lowest priority and 1 is the highest priority)

**Fig. 1:**
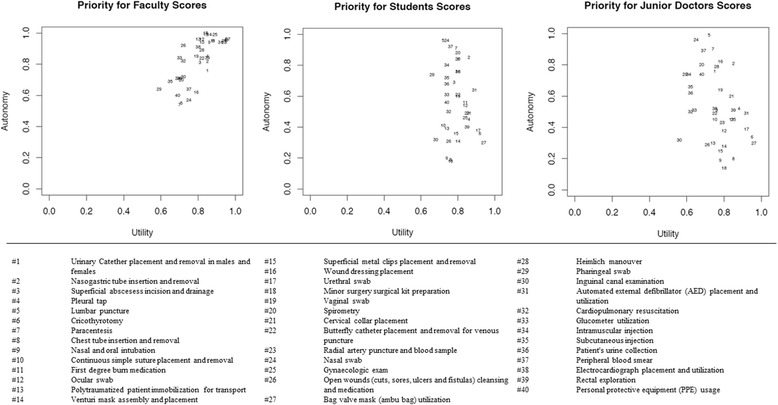
The scatter graphs show the relationship between Autonomy and Utility for Faculty, Students and Junior Doctors

In all three groups, the majority of skills were rated highly utility (the ability to be taught by simulation). Overall rating for utility among the procedures was 7.4/9 for faculty, 7.1/9 for junior doctors, and 7.3/9 for students. Figure 1 gives an overview of the results and their trend; data are all scattered in the right quadrants, which suggests the high level of perceived utility. The lowest utility is 5.9/9 for *Spirometry*, and highest is for *Cardiopulmonary resuscitation* with 8.6/9.

The highest calculated desirability overall was for urinary catheter placement (0.77), nasogastric tube insertion (0.75), and pleural tap (0.70).

Forty respondents (30%) listed additional procedures in the open-ended part of the survey (8 faculty, 16 students, and 16 junior doctors). A total of 76 suggestions were collected. After the exclusion of the non-technical skills (23), duplication (11), and replication (10), 22 new additional procedural skills were suggested (Table 3). Among these, the most often recommended were *first line ultrasounds utilization* (five times), *ear examination* (five times), and *arterial blood pressure measurement* (four times).

**Table 3:** Additional procedural skills suggested by respondents

No.	Procedural skills	No. of times suggested	F	MS	JD
#1	Ear examination	5	0	2	3
#2	First line ultrasounds utilization	5	1	3	1
#3	Blood pressure measurement	4	1	1	2
#4	Earwax removal	3	0	1	2
#5	Drugs dilution	3	0	1	2
#6	Delivery assistance	3	0	0	3
#7	I.V. kit preparation	2	0	0	2
#8	Anterior nasal packing	2	0	0	2
#9	Correct fill-in of medical prescription paper	2	0	0	2
#10	Medical vitals collection	2	2	0	0
#11	Carotid sinus massage	1	0	1	0
#12	Minor surgery	1	0	1	0
#13	Fundus oculi examination without pupil dilation	1	1	0	0
#14	Tracheal aspiration	1	1	0	0
#15	Pediatric BLS	1	1	0	0
#16	Scrotal transillumination	1	1	0	0
#17	Problems-oriented medical chart writing	1	1	0	0
#18	Bone marrow aspiration	1	1	0	0
#19	Small foreign body remotion from the skin	1	0	0	1
#20	Infusion pump management	1	0	0	1
#21	General practitioner software utilization	1	0	0	1
#22	Elastic bandage compression	1	0	0	1
	Total	43	10	10	23

*F* faculty, *MS* graduating medical students, *JD*, junior doctors

## Discussion

To the authors’ knowledge, this is the first study to look at competency development using simulation from a holistic approach; collecting input from faculty, recent graduates, and junior doctors.

Often, one of the barriers to developing a school-wide program is reaching consensus among faculty. In this study, faculty showed a strong general consensus about the importance of the selected procedural skills. Teachers expressed a strong desire that recent graduates be highly proficient to perform all the selected abilities; no ability was rated less than 5.3. In 2007, the Association of American Medical Colleges (AAMC) convened a task-force on clinical skills teaching to provide coherent and broadly applicable model for clinical skills curriculum and performance standards [12]. The identified procedural skills curriculum is similar to our results. Sullivan et al. used a Delphi process to reach consensus among educational leaders at his institution regarding which skills to include in the simulation-based curriculum for all students [13]. Similar attempts are present in literature [3, 14].

The results from student’s self-perception of level of autonomy in performing clinical skill clearly demonstrated a need to establish a structured approach to teach procedural skills at our institution. Students and junior doctors show low self-perceived confidence in many procedural skills. Although there are limitations in the use of self-perceived scores, self-reported level of proficiency or confidence can be an indicator to assess competence [14, 15]. Confidence at carrying out procedures has also been shown to affect performance [16]. Previous studies have identified similar problems with skill acquisition among medical students [3, 17–20] and junior doctors [21–24]. Competency in technical skills is mandatory as deficiency in essential clinical skills could jeopardize the safety of patients [25].

The majority of respondents supported the utility of simulation training for technical skills. Previous studies have demonstrated that the preclinical period is a critical time for providing students a strong foundation in core clinical skills [26]. Bandali et al. have shown that a simulation-enhanced curriculum “successfully bridges the common gap between didactic education and clinical practice.” In addition, learners who have had the opportunity for deliberate practice of skills in a simulation setting have shown to have increased levels of confidence and decreased levels of anxiety when performing skills in the clinical setting [27]. At our institution, students typically learn technical skills by performing supervised procedures on actual patients during clinical rotations. Remmen et al. found that clinical clerkships did not automatically provide an effective learning environment for medical students; clinical rotations are often not adequately focused on technical skills, and students are often passive learners [28].

The desirability function provides an objective metric to prioritize future learning by simulation. By combining the expected level of mastery rated by the faculty, the current level of self-reported competency of the junior doctors and students, and the utility ratings for all groups into a single metric, future simulation activities can be prioritized. For instance, at our institution, urinary catheter placement is the highest priority skill for simulation training; faculty feel that the skill is necessary, the clinical groups did not feel highly competent in the skill, and all groups feel that the skill is highly trainable by simulation. In contrast, personal protective equipment (PPE) usage is low on the priority list; faculty feel they are important, but the junior doctors and medical students are confident in their abilities. Likewise, for cricothyrotomy, although junior doctors and medical students felt that they lacked the skill, faculty gave low importance to acquisitions of the skill, leading to a sixth place priority in the list. In general, desirability functions can be very useful to find an optimal solution in a situation where improvement in one area may be at the expense of another. The function can support decision-making by giving some objectivity to justify such decisions. In this case, the desirability function is designed and implemented to help the organization determine high priority use of simulation to fully developed simulation programs.

The AAMC suggested in 2003 that medical schools be more explicit in the teaching of technical skills [12] Nonetheless, most educational guidelines provide only broad recommendations and do not stipulate the exact technical skills required for graduation. As many educational programs move to competency rather than time-based education, the role of identifying these skills becomes paramount. Although simulation is undoubtedly important for acquisition of these skills, it is only gradually being implemented in most programs. Simulation can be costly, both in technical equipment, and in teaching resources, and it is unlikely most institutions will be able to immediately implement a simulation training program to teach all necessary skills. Use of the survey methodology and the desirability function may serve as a useful tool to prioritize simulation-based technical training. At our own institution, the findings of this study have led to the introduction of a mandatory skills training program in core clinical procedures. It is the hope that this study will prompt similar research within the global framework of medical schools at national and international level.

### Limitations

Although this study describes a methodology that appears to be promising, there are several limitations in the study design. Although the participation rate for the survey was impressive, the study is from a single institution. Priorities will of course vary from site to site, and curriculum development may need to follow the same methodology performed at different sites to find site-specific priorities. In addition, several procedures that were not part of the initial list were added by the study participants: further studies should most likely also include these procedures. The study also relies heavily on the use of self-perceived confidence, which, despite the fact that many studies have shown its utility, must always be considered somewhat biased. A further study conducted using objective measures of competence rather than self-perceived confidence would certainly be useful to validate the present findings. Finally, there are some limitations in the use of the desirability function. Although desirability functions have been in use in industry for decades as a means to compress a multi-dimensional problem to a single metric, there is always a risk of oversimplification inherent in their use. In addition, although in this study, the three components of the overall desirability function were weighted equally, desirability functions can be modified to include differential weighting of the individual components [29].

## Conclusion

Through the use of survey tools this study identified those technical competencies thought by faculty to be important and assessed the junior doctors and recent graduates level of self-perceived confidence in performing these skills. In addition, the study identified how the three groups perceive the utility of teaching these skills by simulation. Finally, the study prioritizes those skills that have a gap between expected and observed competency and are also thought to be amenable to teaching by simulation so that immediate priorities for curriculum simulation development can proceed in the most effective manner.

Although the results of this study are from a single institution, it is hoped that the methodology of survey tools to measure the overall desirability may be useful to researchers and curriculum designers in other centers.

## Abbreviations

AAMC: Association of American Medical Colleges
IQR: Interquartile range
MIUR: Ministry of Education, University and Research
SBME: Simulation-based medical educations

## References

[1] Sičaja M, Romić D, Prka Ž (2006). Medical students’ clinical skills do not match their teachers’ expectations: survey at Zagreb University School of Medicine, Croatia. Croat Med J.

[2] McGaghie WC, Issenberg SB, Cohen ER, Barsuk JH, Wayne DB (2011). Does simulation-based medical education with deliberate practice yield better results than traditional clinical education? A meta-analytic comparative review of the evidence. Acad Med.

[3] Boots RJ, Egerton W, McKeering H, Winter H (2009). They just don’t get enough! Variable intern experience in bedside procedural skills. Intern Med J.

[4] Ministry of Education University and Research. Tabella classi di laurea magistrale. In: Decreto Ministeriale del 16 marzo 2007. http://attiministeriali.miur.it/media/155598/dmcdl_magistrale.pdf. Accessed in Jan 2015.

[5] Vettore L, Tenore A. Presentazione del core curriculum per le abilità pratiche. Medicina e Chirurgia. 2004;24: 955–61

[6] Bandali K, Parker K, Mummery M, Preece M (2008). Skills integration in a simulated and interprofessional environment: an innovative undergraduate applied health curriculum. J Interprof Care.

[7] Berkenstadt H, Erez D, Munz Y, Simon D, Ziv A (2007). Training and assessment of trauma management: the role of simulation-based medical education. Anesthesiol Clin.

[8] Lewis CB, Vealé BL (2010). Patient simulation as an active learning tool in medical education. JMIRS.

[9] McGaghie WC, Issenberg SB, Petrusa ER, Scalese RJ (2010). A critical review of simulation-based medical education research: 2003–2009. Med Educ.

[10] Ziv A, Ben-David S, Ziv M (2005). Simulation based medical education: an opportunity to learn from errors. Med Teach.

[11] Del Castillo E, Montgomery DC, McCarville DR (1996). Modified desirability functions for multiple response optimization. J Qual Technol.

[12] Association of American Medical Colleges. Task Force on the Clinical Skills Education of Medical Students. Recommendations for preclerkship clinical skills education for undergraduate medical education. Washington: Association of American Medical Colleges; 2008. Available at: https://www.aamc.org/download/130608/data/clinicalskills_oct09.qxd.pdf.pdf. Accessed Jan 2015

[13] Sullivan M (2010). The development of a comprehensive school-wide simulation-based procedural skills curriculum for medical students. J Surg Educ.

[14] Fitzgerald JT, White CB, Gruppen LD (2003). A longitudinal study of self-assessment accuracy. Med Educ.

[15] Morgan PJ, Cleave-Hogg D (2002). Comparison between medical students’ experience, confidence and competence. Med Educ.

[16] Byrne AJ, Blagrove MT, McDougall SJ (2005). Dynamic confidence during simulated clinical tasks. Postgrad Med J.

[17] Olajide T, Seyi-Olajide J, Ugburo A, Oridota E (2014). Self-assessment of final year medical students’ proficiency at basic procedures. Maced J Med Sci.

[18] Engum SA (2003). Do you know your students’ basic clinical skills exposure?. Am J Surg.

[19] Ringsted C, Schroeder T, Henriksen J (2001). Medical students’ experience in practical skills is far from stakeholders’ expectations. Med Teach.

[20] Dehmer JJ, Amos KD, Farrell TM, Meyer AA, Newton WP, Meyers MO (2013). Competence and confidence with basic procedural skills: the experience and opinions of fourth-year medical students at a single institution. Acad Med.

[21] Burch VC, Nash RC, Babow T (2005). A structured assessment of newly qualified medical graduates. Med Educ.

[22] Remmen R, Scherpbier A, Derese A (1998). Unsatisfactory basic skills performance by students in traditional medical curricula. Med Teach.

[23] Sachdeva AK, Loicano LA, Amiel GE (1989). Variability in the clinical skills of residents entering training programs in surgery. Surgery.

[24] Sharp LK, Wang R, Lipsky MS (2003). Perceptions of competency to perform procedures and future practice intent: a national survey of family practice residents. Acad Med.

[25] Tekian A (2002). Have newly graduated physicians mastered essential clinical skills?. Med Educ.

[26] Omori DM, Wong RY, Aontonelli MA, Hemmer PA (2006). Introduction to clinical medicine: a time for consensus and integration. Am J Med.

[27] Stewart RA, Hauge LS, Stewart RD (2007). A CRASH course in procedural skills improves medical students’ self assessment of proficiency, confidence and anxiety. Am J Surg.

[28] Remmen R, Denekens J, Scherpbier A, Hermann I, van der Vleuten C, Royen PV (2000). An evaluation study of the didactic quality of clerkships. Med Educ.

[29] Derringer GC (1994). A balancing act: optimizing a product’s properties. Qual Prog.

